# Establishment of a risk assessment system for complications of hepatectomy based on preoperative variables

**DOI:** 10.1016/j.iliver.2022.09.004

**Published:** 2022-10-28

**Authors:** Lining Xu, Guiping Li, Bo Yang

**Affiliations:** a Department of General Surgery, The Second Medical Center & National Clinical Research Center for Geriatric Diseases, Chinese PLA General Hospital, Beijing 100853, China; b Department of Radiology, Hubei Province Integrated Hospital of Chinese and Western Medicine, Wuhan 430015, China; c Department of Radiology, Affiliated Union Hospital, Tongji Medical College, Huazhong University of Science and Technology, Wuhan 430022, China

**Keywords:** Hepatectomy, Complication, Prediction, Preoperative

## Abstract

**Background and aims:**

To reduce the incidence of postoperative complications, it is important to predict them and intervene before surgery if necessary. However, there is no ideal system to evaluate the overall risk of postoperative complications of liver surgery on the basis of preoperative variables. Therefore, this study aimed to design and validate a risk assessment system to predict postoperative complications of hepatectomy on the basis of preoperative variables.

**Methods:**

Binomial logistic regression was used to derive the “hepatectomy overall risk formula” (HORF) for predicting postoperative complications on the basis of preoperative variables.

**Results:**

Multivariate analysis revealed that Child–Pugh grade B–C (odds ratio [OR] = 1.984, *p* = 0.002), medical diseases requiring drug treatment (OR = 1.883, *p* = 0.003), major hepatectomy (OR = 1.947, *p* < 0.001), adjacent organ invasion (OR = 3.616, *p* = 0.023), and preoperative hospital stay >7 days (OR = 1.565, *p* = 0.004) were independent risk factors for postoperative complications of hepatectomy. The area under the curve for the HORF was 0.736. The optimal cut-off value for predicting complications was 0.32 (32%). The area under the curve for the HORF in the validation dataset was 0.727.

**Conclusion:**

The HORF can accurately predict postoperative complications of hepatectomy on the basis of preoperative variables, and thus enables the determination of the necessity for intervention before surgery.

## 1 Introduction

Liver surgery is very difficult to master; it requires continuous and arduous practice [1]. In the past 40 years, breakthroughs in applied basic research, inventions and medical devices, as well as the popularization and improvement of minimally invasive techniques, innovative surgical methods, and diagnostic and treatment standards have led to unprecedented developments in traditional liver surgery [2]. Precision hepatectomy has allowed for significant safety improvements. However, hepatectomy is associated with a high incidence of postoperative complications, which can be fatal [3,4]. Estimating the likelihood of complications before the operation, and intervening at an early stage if necessary, is of clinical importance [5,6]. Although several risk assessment systems have been established, their limitations reduce their clinical utility [7]. As such, there is no ideal system to evaluate the likelihood of hepatectomy complications on the basis of perioperative and preoperative factors. Therefore, a new preoperative evaluation tool improving the safety of hepatectomy would be of great value.

In this study, regression analysis was used to derive a formula for predicting postoperative complications, and an optimal cut-off value was identified. The former should improve the accuracy and objectivity of preoperative evaluations.

## 2 Materials and methods

### 2.1 Patients

We enrolled perioperative patients who underwent hepatectomy and had complete medical records. Patients who recently underwent unexpected reoperations were excluded from the study. A total of 1732 cases were enrolled between January 1986 and December 2020. Seven hundred and thirty one (42.2%) of whom had benign liver diseases, including hepatic hemangioma and hepatolithiasis; and 1001 patients (57.8%) had malignant liver diseases, including intrahepatic cholangiocarcinoma and hepatocellular carcinoma. The median age was 47 years (range: 2–82 years), and there were 1075 (62.1%) men and 657 (37.9%) women. The patients' baseline characteristics are shown in Table 1. In total, 100 patients selected at random from the overall cohort constituted the validation cohort.

**Table 1:** Patient baseline characteristics.

Factors	*n*
General characteristics	
Age (y)	47 (2–82)
Sex	
Female	657 (37.9%)
Male	1075 (62.1%)
Comorbidities	243 (14.0%)
Diagnosis	
Malignant diseases	1001 (57.8%)
Benign diseases	731 (42.2%)
Preoperative evaluation	
Blood test	
Albumin (g/L)	
<35	191 (11.0%)
≥35	1541 (89.0%)
Total bilirubin (μmol/L)	
≤21	1295 (74.8%)
>21	437 (25.2%)
Child–Pugh grade	
A	1552 (89.6%)
B–C	180 (10.4%)
Tumor-related factors	
Largest tumor size (cm)	
≤5	899 (51.9)
5–10	575 (33.2%)
>10	258 (14.9%)
Number of lesions	
One	1505 (86.9%)
Multiple	227 (13.1%)
Operative variables	
Extent of resection	
Minor hepatectomy	967 (55.8%)
Major hepatectomy	765 (44.2%)
Resection type	
Nonanatomical	1201 (69.3%)
Anatomical	531 (30.7%)
Operative duration (min)	
<180	732 (42.3%)
≥180	1000 (57.7%)
Intraoperative blood loss (mL)	
≤800	1516 (87.5%)
>800	216 (12.5%)
Intraoperative blood transfusion	
No	966 (55.8%)
Yes	766 (44.2%)

### 2.2 Data collection

All patients' medical records contained disease history and physical examination data. On the basis of commonly used surgical risk scoring systems and our previous clinical experience, the perioperative factors analyzed were as follows: basic patient characteristics, diagnosis, laboratory examination results, type of surgery, and medical diseases and medication history. Preoperative data included patient demographics, diagnoses, medical diseases medication history, imaging examinations, and laboratory blood analyses (including bilirubin, transaminase, and albumin). Intraoperative data included the extent of resection, estimated blood loss, and operation duration. The postoperative data included hospital length of stay, complications, mortality, and pathology.

### 2.3 Research methods

#### 2.3.1 Risk factors for postoperative complications

Potential independent risk factors for postoperative complications were included in univariate correlation and multivariate logistic regression analyses. Odds ratios (ORs) were calculated, on the basis of which a scoring system was devised. The risk scores were rounded to the nearest integer for clinical applicability. The sum of the scores for all risk factors was defined as the total risk score. The methods are described in more detail in our previous study [8].

#### 2.3.2 “Hepatectomy overall risk formula” for post-hepatectomy complications

Binomial logistic regression of complications was performed, and the hepatectomy overall risk formula (HORF) was calculated as follows: P = 1/{1 + exp [-(α + β_1_χ_1_ + β_2_χ_2_ +···+β_n_χ_n_)]}, where P represents the probability of complications; when P = 1, the probability of complications is 100%. α is a constant term, β_1_–β_n_ are regression coefficients corresponding to the risk index of complications, and χ_1_–χ_n_ are grades for risk factors.

#### 2.3.3 HORF validation

The HORF was used to score and calculate the probability of complications. The receiver operating characteristic (ROC) curve was used to verify the validity of the HORF, and the cut-off value of the risk index was calculated.

### 2.4 Statistical analysis

We used SPSS software (ver. 25.0; IBM Corp., Armonk, NY, USA) for the analysis. Continuous variables, such as estimated blood loss and operation duration, were classified into grades/levels. A *p*-value < 0.05 was considered statistically significant.

## 3 Results

### 3.1 Incidence of hepatectomy complications

Of the 1732 patients, 419 (24.19%) patients developed complications, among whom 164 (39.14%) patients had more than two complications. The complications were fatal in 14 cases (3.34% of all cases with complications), yielding a mortality rate of 0.81% (Table 2).

**Table 2:** Postoperative complications.

Complication	*n*	Ratio relative to the total number of patients
Infection of the abdomen		
Around drainage tube	1	0.06%
Abdominal cavity	1	0.06%
Ascitic fluid	1	0.06%
Intra-abdominal abscess	2	0.12%
Incisional wound	7	0.4%
Peri-liver abscess	22	1.27%
Bile duct		
Sclerosing cholangitis	1	0.06%
Cholangiolitis	5	0.29%
Biliary tract obstruction	1	0.06%
Bile leakage	29	1.67%
Bleeding		
Hemobilia	1	0.06%
Incision bleeding	1	0.06%
Alimentary tract hemorrhage	17	0.98%
Abdominal cavity/raw surface bleeding	13	0.75%
Surgical site-related injuries		
Gastric fistula	1	0.06%
Fistula of anastomosis	3	0.17%
Pancreatic fistula	2	0.12%
Incision disruption	10	0.58%
Wound liquefaction	12	0.69%
Peritoneal effusion		
Pelvic cavity fluid collection	2	0.12%
Retro-peritoneum fluid collection	1	0.06%
Sub-diaphragm fluid collection	6	0.35%
Peri-liver fluid collection (sterile)	90	5.20%
Ascites	129	7.45%
Liver and kidney injury		
Focal hepatonecrosis	1	0.06%
Hepatic inadequacy	26	1.50%
Renal inadequacy	18	1.04%
Pulmonary and cardiovascular		
Pleural cavity infection	1	0.06%
Thrombosis of vena cava	1	0.06%
Heart failure	3	0.17%
Heart infarction	3	0.17%
Hemorrhagic shock	3	0.17%
Respiratory tract infection	5	0.29%
Deep venous thrombosis (lower extremity)	6	0.35%
Pneumothorax	5	0.29%
Respiratory insufficiency	13	0.75%
Atelectasis	26	1.50%
Pneumonia	32	1.85%
Pleural effusion	236	13.63%
Others		
Cerebral accident	2	0.12%
Glottic paralysis	1	0.06%
Electrolyte disturbances	16	0.92%
Dislocation of cricoarytenoid articulation	2	0.12%
Fever of unknown origin	27	1.56%
Ventricular fibrillation	1	0.06%
Peritonitis (sterile)	3	0.17%
Stress ulcer	5	0.29%

### 3.2 Preoperative clinical risk factors for postoperative complications

In univariate correlation analysis, preoperative clinical factors associated with postoperative liver complications included previous history of surgery (*p* = 0.019), history of abdominal surgery history without liver surgery (*p* = 0.011), total bilirubin (*p* < 0.001), medical diseases requiring drug treatment (*p* < 0.001), Child–Pugh grade (*p* < 0.001), extent of resection (*p* = 0.001), pathological type (*p* = 0.006), resection type (*p* < 0.001), adjacent organ invasion (*p* = 0.002), and preoperative hospital stay (*p* = 0.005). These clinical factors were all graded. Since bilirubin is among the factors comprising the Child–Pugh grade, it was combined with the Child–Pugh grade in the evaluation system for simplicity and ease of operation (Table 3).

**Table 3:** Univariate analysis (chi-squared test) of preoperative clinical factors associated with complications of hepatectomy.

Variable	Total (n)	Complications (n)	*p*-value
Previous history of surgery			0.019
Yes	333	97	
No	1399	322	
History of abdominal surgery without liver surgery			0.011
Yes	320	95	
No	1412	324	
Total bilirubin (μmol/L)			<0.001
≤21	1295	255	
>21	437	164	
Medical diseases requiring drug treatment			<0.001
Yes	192	138	
No	1489	281	
Child–Pugh grade			<0.001
A	1552	349	
B–C	180	70	
Extent of resection			0.001
Minor hepatectomy	967	205	
Major hepatectomy	765	214	
Pathological type			0.006
Malignant	951	220	
Benign	682	199	
Resection type			<0.001
Anatomical	531	98	
Nonanatomical	1201	321	
Adjacent organ invasion			0.002
Yes	18	10	
No	1714	409	
Preoperative hospital stay (days)			0.005
≤7	1203	268	
>7	529	151	

Multivariate regression analysis revealed the following independent risk factors for postoperative complications of hepatectomy: Child–Pugh grade B–C (OR = 1.984, *p* = 0.002), medical diseases requiring drug treatment (OR = 1.883, *p* = 0.003), major hepatectomy (OR = 1.947, *p* < 0.001), adjacent organ invasion (OR = 3.616, *p* = 0.023), and preoperative hospital stay >7 days (OR = 1.565, *p* = 0.004) (Table 4). On the basis of the ORs for these factors, a new scoring system was devised. The ORs were rounded to the nearest integer for clinical applicability, as follows: Child–Pugh grade B/C, 2 points; medical diseases requiring drug treatment, 2 points; major hepatectomy, 2 points; adjacent organ invasion, 4 points; preoperative hospital stay >7 days, 2 points (Table 5).

**Table 4:** Results of logistic regression analysis identifying factors independently influencing post-hepatectomy complications.

Variable	Odds ratio	*p*-value	Score
χ_1_：Child–Pugh grade (A/B–C)	1.984	0.002	1/2
χ_2_：Medical diseases requiring drug treatment (no/yes)	1.883	0.003	1/2
χ_3_：Extent of resection (minor hepatectomy/major hepatectomy)	1.947	<0.001	1/2
χ_4_：Adjacent organ invasion (no/yes)	3.616	0.023	1/4
χ_5_：Preoperative hospital stay (≤7/> 7 days)	1.565	0.004	1/2

**Table 5:** Components of the new scoring system.

Variable	Level	Score
χ_1_：Child–Pugh grade	A	1
	B–C	2
χ_2_：Medical diseases requiring drug treatment	No	1
	Yes	2
χ_3_：Extent of resection	Minor hepatectomy	1
	Major hepatectomy	2
χ_4_：Adjacent organ invasion	No	1
	Yes	4
χ_5_：Preoperative hospital stay (days)	≤7	1
	>7	2

Note: the total score was calculated by summing the scores for the five individual variables.

### 3.3 Establishment of a formula for calculating the risk of complications of hepatectomy

A binomial logistic regression model was established with the risk scores (for hepatectomy complications) of preoperative factors as independent variables and occurrence of complications as the dependent variable. The intercepts (regression coefficients) are shown in Table 6. The formula for calculating the risk of complications was as follows:

P = 1/{1 + exp [-(-4.844 + 0.737χ_1_ + 0.530χ_2_ + 0.673χ_3_ + 0.412χ_4_ + 0.464χ_5_)]}

**Table 6:** Logistic regression coefficients of factors associated with hepatectomy complications.

Variable	Intercept	Wald statistic
χ_1_：Child–Pugh grade (B–C/A)	0.737 (β_1_)	14.530
χ_2_：Medical diseases requiring drug treatment (no/yes)	0.530 (β_2_)	7.235
χ_3_：Extent of resection (minor hepatectomy/major hepatectomy)	0.673 (β_3_)	26.759
χ_4_：Adjacent organ invasion (no/yes)	0.412 (β_4_)	5.299
χ_5_：Preoperative hospital stay (≤7/> 7 days)	0.464 (β_5_)	11.809
Constant	−4.844(α)	144.346

### 3.4 Predictive ability of the combined variables

**Fig. 1:**
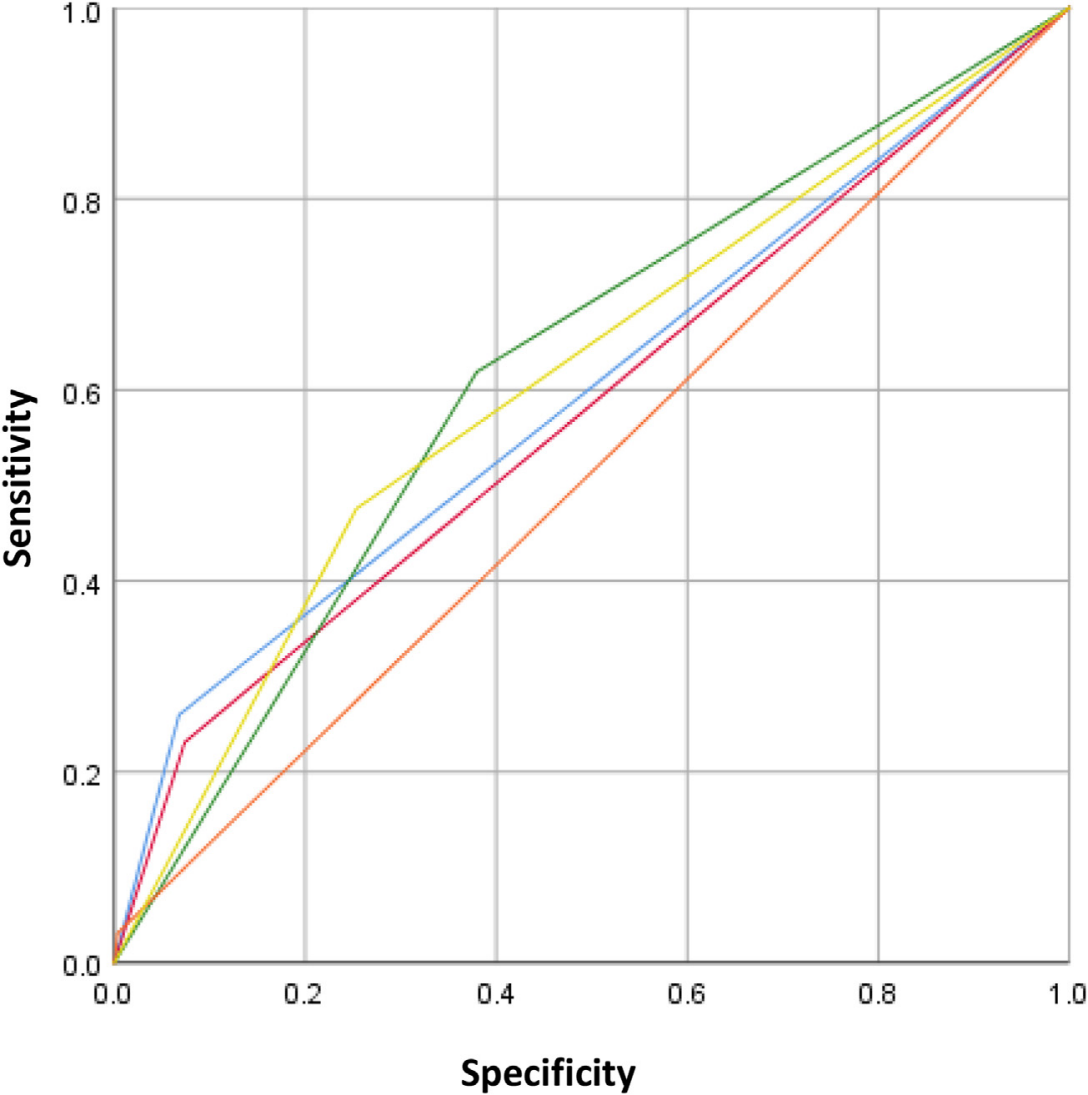
Predictive ability of the five variables. Blue line, Child–Pugh grade; purple line, medical diseases requiring drug treatment; green line, number of segments resected; red line, adjacent organ invasion; yellow line, preoperative hospital stay. The area under the curve values for postoperative complication were 0.596, 0.579, 0.620, 0.514, and 0.611 for Child–Pugh grade, medical diseases requiring drug treatment, extent of resection, adjacent organ invasion, and preoperative hospital stay, respectively.

**Fig. 2:**
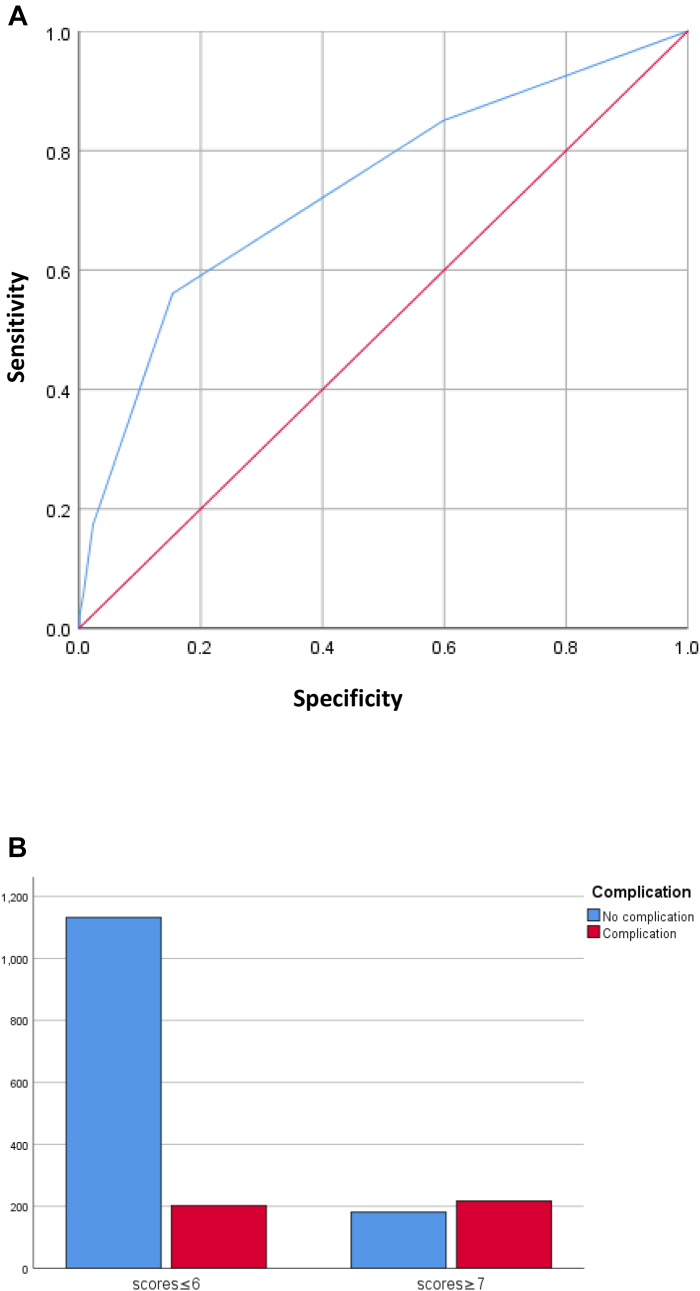
(A) Predictive ability of the combined variables (area under the curve = 0.735). (B) Incidence of complications. The incidence of complications in the group with ≤6 points on the new scoring system was significantly lower than that in the group with ≥7 points (*p* < 0.001).

The ROC curves for each factor are plotted in Fig. 1. The ROC curve of the five variables combined is shown in Fig. 2A; the area under the curve (AUC) was 0.735 (standard error = 0.015). The total score was calculated by summing the scores for all factors. The optimal cut-off total score for postoperative complications was 6.5; the incidence of postoperative complications was significantly different between groups with ≤6 and ≥7 points (χ^2^ = 59.217, *p* < 0.001), as shown in Fig. 2B.

### 3.5 Predictive ability of the HORF

**Fig. 3:**
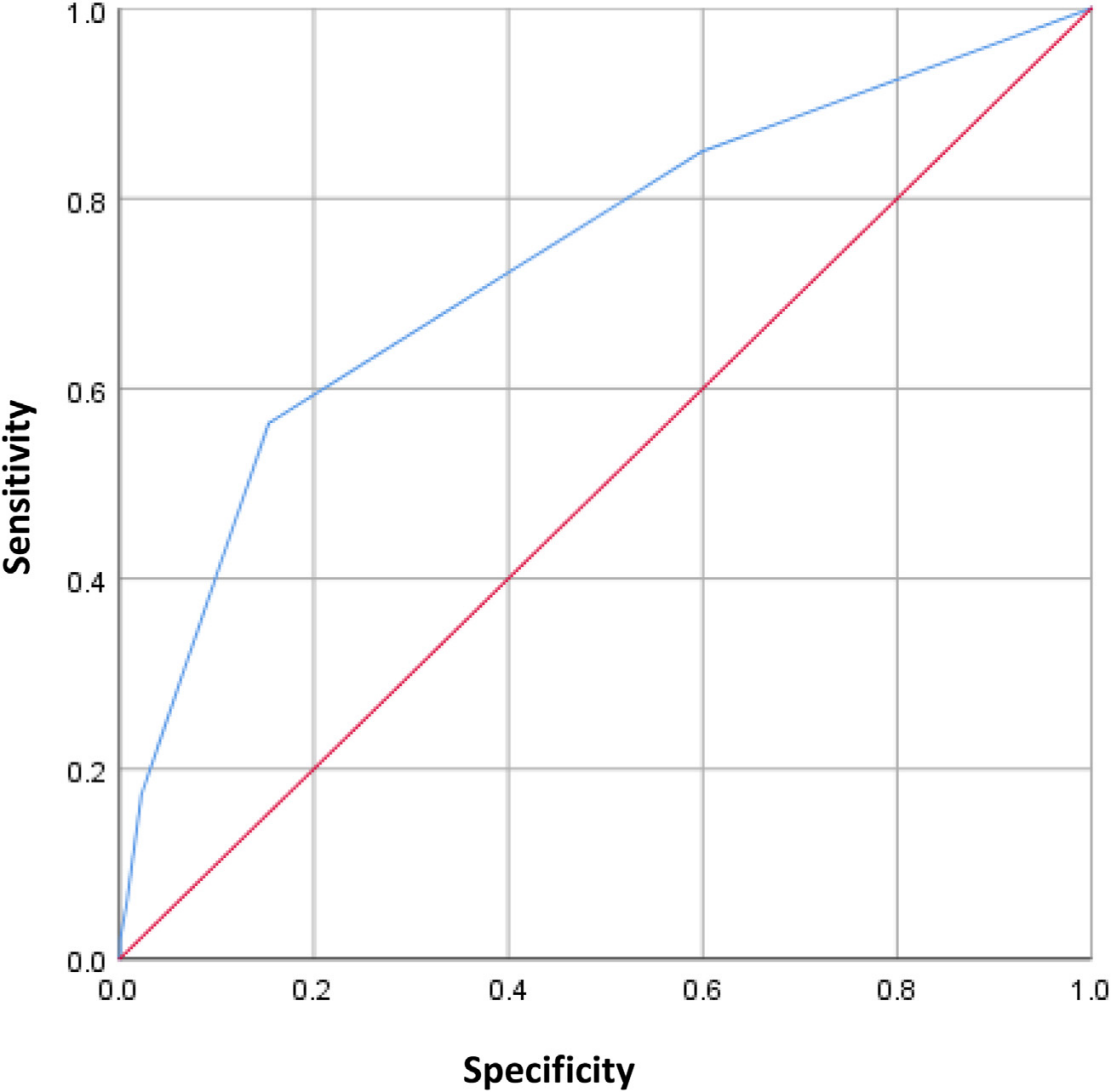
Predictive ability of the hepatectomy overall risk formula (HORF). The area under the curve for the HORF was 0.736.

The scores for each variable and patient were substituted into the HORF to calculate the expected probability of complications, and an ROC curve was generated to evaluate the predictive ability, as shown in Fig. 3. The AUC of the HORF was 0.736 (standard error = 0.016). The optimal cut-off value for predicting complications was 0.32 (32%).

### 3.6 Validation of the HORF

**Fig. 4:**
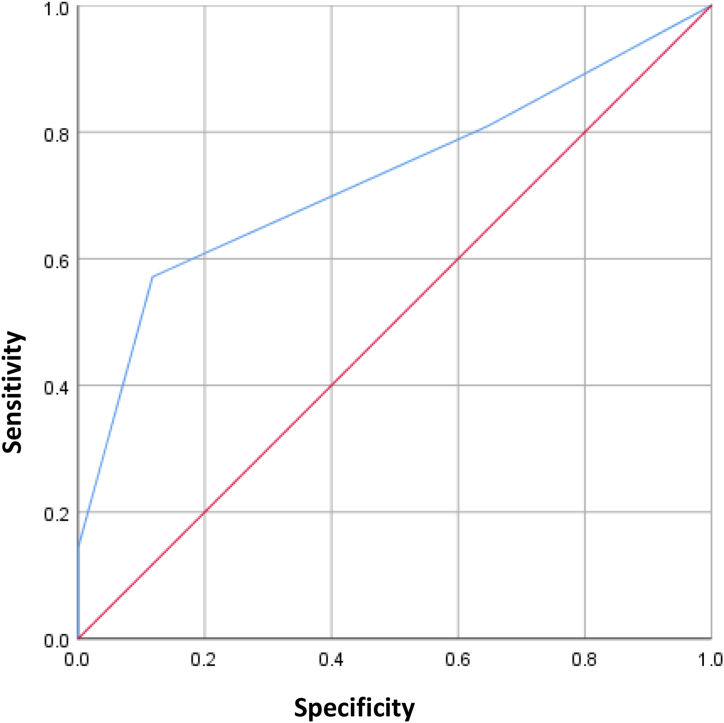
Validation of the hepatectomy overall risk formula (HORF). The area under the curve of the HORF was 0.727.

An ROC curve analysis was used to validate the HORF. The AUC of the HORF was 0.727, as shown in Fig. 4.

### 3.7 Study flow chart

**Fig. 5:**
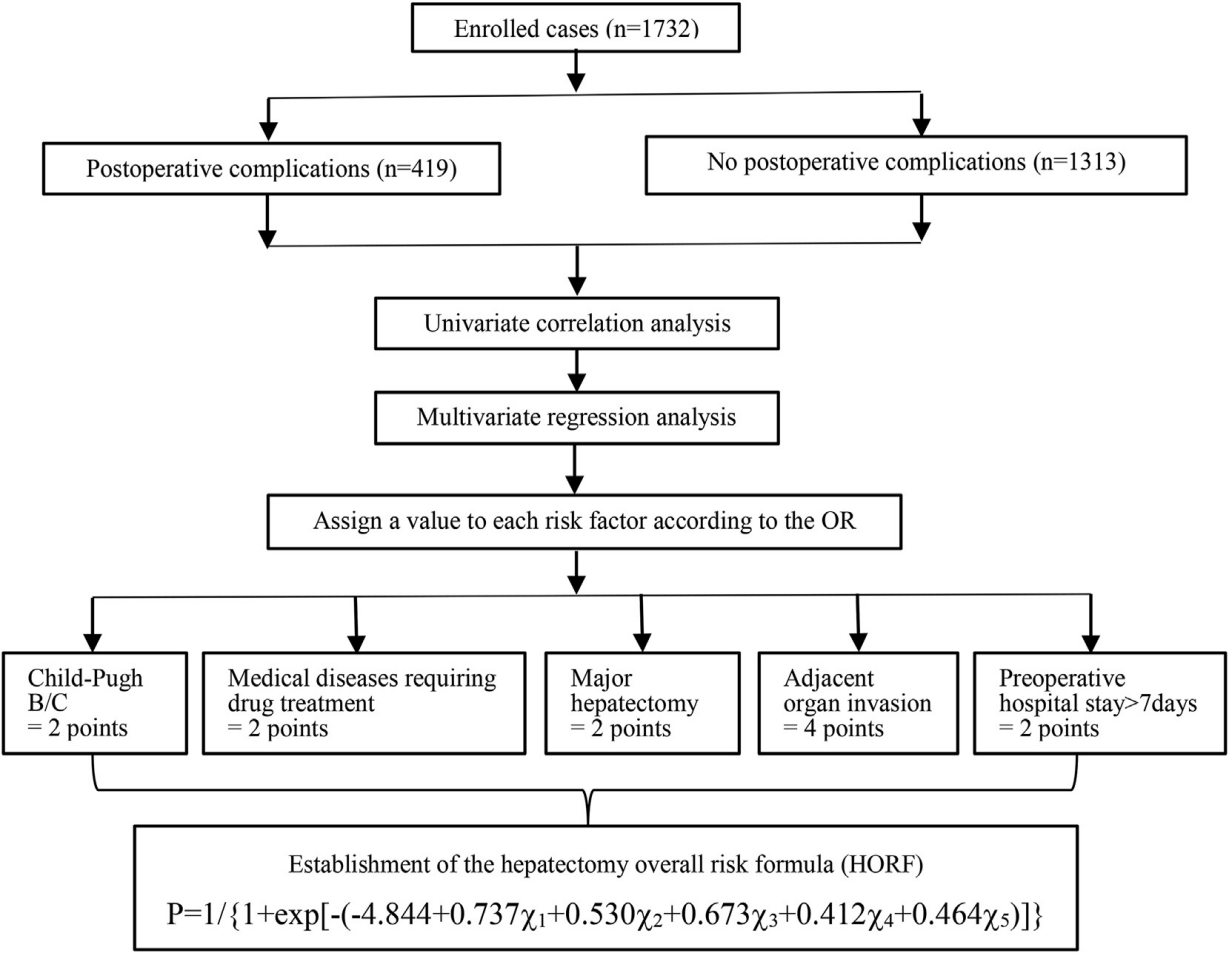
Study flow chart.

A flowchart of this study is shown in Fig. 5.

## 4 Discussion

The safety of hepatectomy has always been an important consideration in liver surgery [9]. The morbidity and mortality rates of hepatectomy remain high, and improving preoperative prevention strategies to reduce the incidence of postoperative complications is a difficult task [10]. Unfortunately, there is still no intuitive and accurate system to predict hepatectomy complications on the basis of preoperative factors [11]. Therefore, in this study, we established a preoperative risk prediction and evaluation system for hepatectomy complications to aid interventions in high-risk patients before surgery, and thus improve the safety of liver surgery.

Liver failure after hepatectomy is an important cause of death [12]. Precise assessment of liver function is crucial for hepatectomy, where the balance between the operative procedure and remnant liver function is the most important concern for patients with chronic liver disease [13]. The Child–Pugh grade is the most widely used method for evaluating liver function [14]. In this study, the Child–Pugh grade was an independent risk factor for complications after hepatectomy. However, due to the subjectivity of some indicators, the Child–Pugh grade alone is insufficient to accurately predict postoperative liver complications; other indicators should also be used.

Many patients who require surgery have one or more additional medical conditions, termed comorbidities [15]. Comorbidities, which are particularly common in elderly individuals, can affect disease manifestations and severity, and sometimes impact management [16]. Old age and its attendant risks are the major predisposing factors for poor postoperative outcomes [17]. With the development of traditional surgery, a large number of high-risk surgery patients with single or multiple organ dysfunctions have undergone surgery, and the number of surgical patients with atherosclerosis, diabetes, chronic obstructive pulmonary disease, and other internal diseases has increased rapidly. In this study, concomitant medical condition requiring medication was an independent risk factor for postoperative complications of hepatectomy. Although the rate of comorbidities gradually increased with age in our cohort, our results do not suggest that age is related to complications. The incidence of complications was low among the elderly surgical patients without comorbidities, indicating that comorbidities was the decisive factor in terms of complications, rather than age.

Giant liver tumors, which were considered inoperable in the past, have been safely resected in recent years, mainly due to improvements in surgical techniques and perioperative management [4]. For an optimal perioperative strategy, the measurement of vital signs, maintenance of electrolyte balance and normal function of vital organs, and intraoperative application of aseptic dressings, hemostatic dressings, and sutures is essential. However, the amount of hepatectomy (residual functional liver volume) remains the most important factor in terms of liver dysfunction after hepatectomy [18]. This study showed that more extensive resection was an independent risk factor for complications after hepatectomy. Similarly, many studies have shown that the more extensive the operation, the more likely it is that complications will occur. Among our patients with extrahepatic organ invasion, the risk of complications was greater when organ resection was performed simultaneously.

The length of the preoperative hospital stay was correlated with illness severity in this study; the more severe the illness, the more complicated the preoperative preparation and the longer the hospital stay. In contrast, in uncomplicated cases with few comorbidities, preoperative preparation is simple and short. In this study, multivariate analysis showed that preoperative hospital stay was independently associated with postoperative complications of liver surgery.

Chen et al. [11] established a simplified scoring system based on perioperative parameters including diabetes, the extent of surgery, the serum potassium level, and blood loss relative to body weight. However, blood loss is an intraoperative measure that can only be assessed at the end of the operation, such that their scoring system cannot be used to evaluate the risk of postoperative complications preoperatively. Our HORF can predict the occurrence of postoperative complications using only preoperative indexes, and thus provides a basis for preoperative intervention. Donadon et al. [19] established the Humanitas score for individualized risk estimation of postoperative morbidity after hepatectomy. This scoring system includes the following indicators: major hepatectomy, liver stiffness, the BILCHE score, and esophageal varices. However, as a limitation, liver stiffness is not typically examined in the clinical setting; some hospitals do not perform this examination, which limits the applicability of this scoring system. Similarly, the BILCHE score is not often used in clinical practice, and the clinical utility of cholinesterase is limited compared with the Child–Pugh grade. Finally, the diagnosis of esophageal varices can only be confirmed by upper gastrointestinal endoscopy, which is complicated and causes great discomfort to patients.

Some limitations of this study should also be noted. First, due to limitations in terms of the researchers, case screening, and available time, among other factors, the HORF was subjected to internal validation rather than the more powerful external validation. However, due to the large sample size, the validation results can be considered reliable. We are planning future research to further refine the HORF.

In this study, a binomial logistic regression model was established to obtain the HORF, which allows assessment of the risk of complications of hepatectomy. As a novel and simple assessment system, the HORF can be used to effectively predict postoperative complications of hepatectomy before surgery, i.e., on the basis of preoperative factors.

## Funding

This study was supported by the General Project of 10.13039/501100003819Natural Science Foundation of Hubei Province (2020CFB826).

## Author contributions

Xu L, Li G, and Yang B designed the research, performed the primary literature search, and extracted the data. Xu L analyzed the data, Yang B wrote the manuscript, and Xu L, Li G, and Yang B were responsible for revising the manuscript for important intellectual content. All authors read and approved the final version of the manuscript.

## Acknowledgments

The authors thank Mrs. Guang Yang for her assistance with the data collection.

## Declaration of competing interest

The authors declare that they have no known competing financial interests or personal relationships that could have appeared to influence the work reported in this paper.

## Data available statement

The patient data that support the findings of this study are available on request from the corresponding author. The data are not publicly available due to privacy or ethical restrictions.

## Ethics statement

This study was approved by the ethics committee of Chinese PLA General Hospital. All patients provided informed consent. This study was performed according to the ethical standards of the Declaration of Helsinki.

## Informed consent

This study used deidentified patient information and was conducted in accordance with the principle of informed consent.
